# Concordance of self- and informant-rated depressive symptoms in nursing home residents with Dementia: cross-sectional findings

**DOI:** 10.1186/s12888-022-03876-5

**Published:** 2022-04-05

**Authors:** Julie L. O’Sullivan, Roxana Schweighart, Sonia Lech, Eva-Marie Kessler, Christina Tegeler, Andrea Teti, Johanna Nordheim, Paul Gellert

**Affiliations:** 1 Institute for Medical Sociology and Rehabilitation Science, Charité Universitätsmedizin Berlin, corporate member of Freie Universität Berlin, Humboldt-Universität Zu Berlin, and Berlin Institute of Health, Charitéplatz 1, 10117 Berlin, Germany; 2 Institute for Gerontology, University Vechta, Driverstraße 2, 49377 Vechta, Germany; 3 Department of Psychiatry and Psychotherapy, Charité – Universitätsmedizin Berlin, corporate member of Freie Universität Berlin, Humboldt-Universitätzu Berlin, and Berlin Institute of Health, Charitéplatz 1, 10117 Berlin, Germany; 4 Department of Psychology, MSB Medical School Berlin, Rüdesheimer Str. 50, 14197 Berlin, Germany

**Keywords:** Depression screening, Neuropsychiatric symptoms, Dementia, Proxy-rating, Quality of life, Geriatric mental health

## Abstract

**Background:**

Depression is highly prevalent in nursing home residents living with moderate to severe dementia. However, assessing depressive symptoms in residents with dementia can be challenging and may vary by rater perspective. We aimed to investigate the concordance of, and factors associated with self- and informant-rated depressive symptoms in nursing home residents with dementia.

**Methods:**

Cross-sectional data was collected from *N* = 162 nursing home residents with dementia (age: 53–100; 74% women). Self-ratings were assessed with the Geriatric Depression Scale, while the depression and anxiety items of the Neuropsychiatric Inventory were used for informant-ratings. Cohen’s Kappa was calculated to determine the concordance of both measures and of each with antidepressant medication. Multivariate associations with sociodemographic variables, self- and informant-rated quality of life, dementia stage, neuropsychiatric symptoms, functional status and antidepressant medication were analysed with linear mixed models and generalized estimating equations.

**Results:**

Concordance between self- and single item informant-rated depressive symptoms was minimal (Cohen’s Kappa = .22, *p* = .02). No concordance was found for self-reported depressive symptoms and the combined informant-rated depression-anxiety score. Self-reported depression was negatively associated with self-rated quality of life (β = -.32; 95%CI: -.45 to -.19, *p* < .001), informant-rated quality of life (β = -.25; 95%CI: -.43 to -.07, *p* = .005) and functional status (β = -.16; 95%CI: -.32 to -.01, *p* = .04), whilst single item informant-rated depression revealed negative associations with informant-rated quality of life (β = -.32; 95%CI: -.52 to -.13, *p* = .001) and dementia stage (β = -.31; 95%CI: -.52 to -.10, *p* = .004). The combined informant-rated depression-anxiety score showed negative associations with self-rated quality of life (β = -.12; 95%CI: -.22 to -.03, *p* = .01) and dementia stage (β = -.37; 95%CI: -.67 to -.07, *p* = .02) and a positive association with neuropsychiatric symptoms (β = .30; 95%CI: .10 to .51, *p* = .004). No concordance was found with antidepressant medication.

**Conclusions:**

In line with our expectations, low agreement and unique association patterns were found for both measures. These findings indicate that both instruments address different aspects of depression und underline the need for comprehensive approaches when it comes to detecting signs of clinically relevant depressive symptoms in dementia.

**Trial registration:**

The trial was registered with the ISRCTN registry (Trial registration number: ISRCTN98947160).

## Introduction

Although cognitive and functional decline are considered hallmarks of dementia, the vast majority of people living with dementia (PWD) also experience at least one neuropsychiatric symptom (NPS) in the course of the disease [1, 2] and NPS are especially prevalent in nursing home residents with dementia [3]. Depression is among the most frequent NPS in dementia with reported prevalence rates ranging from 20 to 60% [4, 5]. Depressive symptoms are known to cause distress, decrease quality of life and exacerbate cognitive and functional impairments in PWD [6]. However, depression often goes undiagnosed and therefore untreated in PWD living in nursing homes [7–9], even though higher prevalence of depression has been reported for nursing home residents with dementia compared to residents without dementia [10, 11] and to community-dwelling PWD [12]. 

The relationship between dementia and depression is complex [13]. Depressive symptoms occurring in later life are a known risk factor for the presence of cognitive deficits and dementia [14], however late onset of depressive symptoms can also constitute an early manifestation of dementia [15]. In turn, dementia is a risk factor for depression due to psychological reaction to the cognitive and behavioral changes accompanying dementia [16]. Comorbid depression in dementia is associated with a profound decrease in quality of life [17], accelerated cognitive decline [18], increased mortality [19] and caregiver stress [6]. Therefore, the diagnosis and treatment of depression in PWD should be a clinical priority, especially with regard to quality of life of PWD and their informal caregivers [6].

There are several appropriate therapeutic options for the treatment of depressive symptoms in PWD, including mainly non-pharmacological interventions, such as interpersonal psychotherapy, cognitive behavioral therapy, reminiscence therapy, structured activity programs, and sensory stimulation therapy [6, 20, 21]. Pharmacological treatment with antidepressants is recommended for PWD in some cases of severe and persistent depressive symptoms, although there is limited evidence of benefit [22]. Thus, with an increased risk of side effects, non-pharmacological interventions should be the preferred option for treatment of depression in PWD [21].

Accurate diagnosis of depression is essential in order to initiate appropriate interventions. It has been argued that numerous disease, clinician and system-level factors may hinder the diagnosis of depression in PWD [23, 24]. Insufficient diagnosis of depression may also lead to inappropriate use of antidepressants, which has been reported in nursing home residents with and without dementia [25, 26]. In this regard, Kramer et al. [27] found low concordance of depression diagnosis and prescriptions of antidepressants in PWD, similar findings have been reported by Kwak et al. [28]. Moreover, Wetzels et al. [29] found a negative association of antidepressant medication with quality of life in PWD. The concept quality of life is closely linked to depression in PWD, for instance as the presence of major depression was found to be the most important risk factor for poorer quality of life in nursing home residents with dementia [30]. A systematic review by Beerens et al. [17] further highlights the negative impact of depressive symptoms on quality of life in PWD.

While structured clinical interviews remain the goldstandard for diagnosis of depressive symptoms in dementia, self- and informant-rated assessments scales are usually applied for assessment of depressive symptoms in PWD under the real-world conditions of long-term care facilites [21]. Although informant-ratings can be useful to objectify PWDs’ functional and cognitive status [31], they have been criticized for their paternalistic approach [32] and potential lack of validity for assessing more subjective outcomes such as well-being [33] and quality of life in PWD [34, 35]. Regarding depressive symptoms, few studies have investigated the agreement between self- and informant-ratings of depressive symptoms in PWD and it remains unclear, whether and how information from these two modalities actually gives a consistent picture [36]. Arlt et al. [37] found good congruence of clinician-rated depressive symptoms assessed with the Montgomery Asberg Depression Rating Scale (MADRS) [38] and self-reports assessed with the Geriatric Depression Scale (GDS) [39] in a cross-sectional study with mostly community-dwelling PWD. Regarding self- and informant-ratings of depressive symptoms in PWD in nursing homes, one study reported moderate agreement using the Cornell Scale for Depression in Dementia (CSDD) [40] for both self- and informant-ratings [41]. In contrast however, two previous studies compared self-ratings of depression using the GDS with informant-ratings using the depression item of Neuropsychiatric Inventory (NPI) [42] and found low agreement of both measures in community-dwelling people with mild dementia [28, 36]. However, a review of 15 studies investigating the factor structure of the NPI showed that some studies found a “mood” or “affective” factor composed of the depression item and the anxiety item of the NPI, indicating the existence of an “affective”-subsyndrome [43]. Thus, one could argue that this subsyndrome may be conceptually more closely linked to depressive symptoms according to GDS. In summary, these conflicting findings may be related to differences in the applied assessment tools and/or study populations, i.e., levels of dementia severity.

Knowlegde is sparse about associations of self- and informant-depression ratings with other patient and context-related factors in nursing home residents with dementia. Gruber-Baldini et al. [44] investigated predictors of staff-rated depression in long-term care and found associations with disease-related factors, i.e., severe cognitive impairment, behavioral symptoms, and pain, as well as living in for-profit nursing homes. In a cross-sectional study with community-living PWD, Dawson et al. [45] found self-reported depression was predicted by physical strain and role captivity. To our knowlegde, factors associated with both self- and informant-ratings of depressive symptoms in nursing home residents with moderate to severe dementia have not been investigated in one and the same study. However, a systematic review including ten cross-sectional and three longitudinal studies on characteristics of self- and informant-ratings of quality of life, a concept closely linked to depression in PWD living in long-term care facilities, revealed disparate association patterns within and across studies [17]. Taken together, the concordance of self- and informant-rated depressive symptoms in PWD living in nursing homes and their specific association patterns with other constructs need further investigation.

### The present study

The aim of this cross-sectional study was (1) to determine the concordance between self- and informant-rated depressive symptoms in nursing home residents living with dementia (2) to investigate factors associated with both modalities and (3) to assess concordance of self- and informant-rated depressive symptoms with antidepressant medication. Based on previous findings, we expect only moderate concordance between self- and informant-rated depressive symptoms. However, we expect the concordance with self-rated depressive symptoms will improve for a global informant-rated mood score, as indicated by a combined depression-anxiety score compared to a single item informant-rated score. We further expect self-rated depressive symptoms to be associated with subjective factors such as functional ability, meaning the capacity to carry out activities of daily living independently and self-reported quality of life, whilst expecting informant-rated depressive symptoms to be associated with caregiver and disease-related factors such as severity of NPS, dementia stage and informant-rated quality of life. Furthermore, low concordance of depressive symptoms with antidepressant medication is expected.

## Materials and methods

### Participants and recruitment

The present study took place within the scope of the research project CareTab (German language: *PflegeTab*). The primary objective of CareTab was to investigate the effects of a tablet-based intervention for nursing home residents with moderate to severe dementia in a cluster-randomized controlled trial (cRCT). The main results of the trial have been published elsewhere [46]. In the present paper, we report cross-sectional analyses of baseline data collected in ten nursing homes located in Berlin, Germany. All participants were long-term residents of the included nursing homes. As the focus of the CareTab study was on nursing home residents with dementia, only residents with a pre-existing dementia diagnosis were screened for eligibility. In nine participating facilities, all residents with dementia were screened. The remaining facility had a special dementia unit, in this case only PWD from this unit were included in the further recruitment process. Inclusion criteria were dementia diagnosis or cognitive impairment meaning a Mini-Mental State Examination (MMSE) [47] score of less than 24 points. Exclusion criteria were pre-existing severe mental and behavioural disorders other than depression and dementia, and short-term residency of less than four weeks. Legal guardians of eligible PWD were first contacted by telephone. Upon their written consent, PWD were approached and thoroughly informed about the study. Comprehensive verbal and written information was provided for both PWD and guardians about the research project and the trial. The CareTab study was conducted in accordance with the Declaration of Helsinki and was reviewed and approved by the Ethics Committee of the Medical University of Berlin (Charité – Universitätsmedizin Berlin; EA1/013/16) and registered with the ISRCTN registry (Trial registration number: ISRCTN98947160).

### Measures and procedure

Two trained and experienced research assistants visited each nursing home and collected self- and informant-rated data on site. Each participant was interviewed once by either one of the research assistants. Self-rated assessments were conducted as interviews directly with PWD. The research assisstants were instructed to approach all participating PWD to obtain self-reported data, regardless of their dementia stage. However, self-reports were not to be continued if PWD declined or if they seemed overwhelmed or distressed by the interview questions. Informant data on participants was assessed from members of the nursing home staff (trained nursing professionals) who had worked with the participant on a regular basis and thus knew them well. Informant data was also collected once by either of the research assistants.

### Depressive symptoms

*Self-reports of depressive symptoms* were measured using the GDS-15 [39], which is a 15-item questionnaire in a yes/no format. Scores range from 0–15, higher scores indicate a higher risk of depression. The GDS is one of the most widely used depression screening instruments in older populations and the 15-item version is recommended in nursing home settings [48]. With reliability values ranging from 0.88–0.89 across different studies [49, 50], the GDS-15 is considered a highly reliable instrument. A recent meta-analysis found a pooled sensitivity and specificity of 86% and 79% for the 15-item GDS version, with higher diagnostic accuracy than the longer 30-item version [51]. According to Arlt et al. [37] scores of 0–4 indicate no clinical depression, scores of 5–8 indicate mild depression, scores of 9–11 indicate moderate depression and scores of 12–15 indicate severe depression. Although the GDS-15 is not recommended for people with severe dementia [52], we approached all participants in an attempt to allow them to give self-ratings if possible. The depression item of the Neuropsychiatric Inventory – Nursing Home version (NPI-NH) [42] was used to measure *informant-rated depressive symptoms*. The NPI-NH is a screening instrument frequently used in the nursing field to evaluate NPS in PWD. The sufficient reliability and validity postulated in the original instrument [42] has been confirmed in studies analysing psychometric properties for the Nursing Home Edition. Thus, reliabilities between 0.64–0.80 with sufficient validity have been reported [53, 54]. This information is roughly congruent with that of the original version, which assumes an overall reliability of 0.88 and acceptable validity for the NPI [42]. Trained nursing professionals are first asked a screening item consisting of three questions to determine if depression is present or not: “Does the resident seem sad or depressed? Does he/she say that he/she feels sad or depressed? Does the resident cry at times?” If any screening question is answered with “yes”, subquestions are asked to confirm the presence of depression. If depression is confirmed, informants are asked to rate frequency and severity of depression. Possible scores range from 0 (no sign of depression) to 12 (frequent and severe signs of depression) and are computed by multiplying severity (1 = mild – 3 = severe) by frequency (1 = rarely – 4 = very often). A composite score combining the NPI-NH depression and anxiety items was also computed in order to assess associations of the informant–rated depression-anxiety subsyndrome of the NPI-NH with other study variables.The screening questions for anxiety are: “Is the resident very nervous, worried, or frightened for no reason? Does he/she seem very tense or unable to relax? Is the resident afraid to be apart from you or from others that he/she trusts?”. The scoring procedure is analogous to the depression item. Scores of the depression and anxiety items were summed to calculate the combined depression-anxiety score. Both the NPI-NH and GDS-15 were chosen because they are among the most frequently applied instruments in nursing homes [6].

### Other study variables

*Self-rated quality of life* was measured with the Quality of Life in Alzheimer's Disease (QOL-AD) questionnaire in PWD who were able to respond to the questions. Within the 13-item QOL-AD, participants are asked to rate different aspects of their lives on a 4-point Likert scale, total scores range from 13–52. Additionally, *informant-rated quality of life* was assessed with the more comprehensive QUALIDEM scale [55]. QUALIDEM consists of 37 items belonging to nine subscales. All items are rated on a 4-point Likert scale, total scores range from 0–111. In accordance with previous studies, we computed a total score for the QUALIDEM scale [56]. Higher scores reflect higher quality of life levels in both measures. *Dementia stage* was measured with the Functional Assessment Staging (FAST), which consists of 7 major functional stages and 11 substages. Stage levels increase as dementia progresses with stage 4 corresponding to mild dementia, stage 5 to moderate, stage 6 to moderately severe, and stage 7 to severe dementia. [57]. *Functional status* was assessed with the Barthel Index [58]. Scores range from 0–100. Higher Scores reflect higher functional ability. *Neuropsychiatric symptoms* were assessed with the informant-rated NPI-NH, which evaluates 12 NPS commonly observed in PWD using standardized interview questions. Nursing professionals are asked to rate the frequency and severity of each neuropsychiatric symptom. Scores are computed for each symptom by multiplying severity (1 = mild – 3 = severe) by frequency (1 = rarely – 4 = very often). Higher scores represent higher degrees of NPS. As we analysed the single depression item of the NPI-PH and the combined depression-anxiety score separately, composite scores for neuropsychiatric symptoms were computed by adding up the scores of the remaining 11 or 10 NPI-NH subscales. We assessed the intake of *antidepressant medication* by examining patient records. The presence of antidepressants was coded as 0 = not present or 1 = present and information was taken from medical records of the facilities. The PRISCUS list, the German equivalent of the Beers list, was used to assess potentially inadequate antidepressants [59].

### Statistical analysis 

Descriptive statistics are reported as means and standard deviations for continuous variables, and as absolute and relative frequencies for categorical variables. Frequency of missing data was below 5% for informant-rated measures. Missing data occurred at item and scale level on all self-reported measures. Values on self-reported depressive symptoms (GDS-15) were substituted with the mean of an individual participants' non-missing items if the majority of scale items (≥ 8) were available. For self-reported quality of life scores (QOL-AD), total scores were computed for partcipants with a maximum of two missing items, as recommended by Logsdon, Gibbons [60]. Distribution of the data was not normal, therefore non-parametric Mann–Whitney U Tests were used to compare participants with and without self-ratings of depressive symptoms. We calculated Spearman’s correlation coefficient and Cohen’s Kappa statistic to determine association and agreement between self- and informant-rated screening instruments of depressive symptoms and antidepressant medication [61]. Linear mixed-effects models (LMM) fit by Restricted Maximum Likelihood Estimation were used to investigate associations of self- and informant-rated measures of depressive symptoms with other study variables. Generalized estimating equations (GEE) were used when more robust estimation methods lead to more stable models. Cases with incomplete data were omitted from the GEE models, leading to smaller sample sizes in these analyses. Further variables (i.e., age, gender, self- and informant-rated quality of life, dementia stage, functional status, neuropsychiatric symptoms, antidepressant medication) were included as fixed covariates and a random intercept was added at nursing home-level to account for clustering of participants in nursing homes. All covariates were added to a multivariate model. Included variables were standardized to allow meaningful interpretation of coefficient estimates as β coefficients. All statistical analyses were performed using IBM SPSS statistics software (IBM SPSS Statistics for Windows, Version 27.0. Armonk. NY: IBM Corp). All tests of significance were based on a *p* < 0.05 level and confidence interval of 95%.

## Results

A total of *N* = 162 nursing home residents with dementia were included in the study. The mean age was 85.0 years (SD = 7.1) and 74% were women. According to the informant-rated FAST score, 135 participants (84%) were classified as living with moderately severe dementia (FAST stage 6) and mean functional status score (Barthel Index) was 53.6 (SD = 26.2), indicating severe dependency. Table 1 shows an overview of participant characteristics.

**Table 1:** Participant characteristics, *N* = 162

Characteristic	*n*	
Demographics		
Age (years), M (SD)	162	85.0 (7.1)
Women, n (%)	162	119 (74)
Care level, n (%)	158	
Minor impairment		0 (0)
Substantial impairment		2 (1)
Serious impairment		51 (32)
Most severe impairment		83 (53)
Most severe impairment wto special care needs		22 (14)
Cognitive Status		
MMSE, M (SD)	113	14.4 (6.5)
Type of Dementia, n (%)	154	
Alzheimer’s Disease		29 (19)
Vascular Dementia		15 (10)
Unspecified Dementia		77 (50)
Mixed Dementia		17 (11)
Others		16 (10)
Depressive Symptoms		
Self-rated GDS-15, M (SD)	121	3.8 (3.0)
Informant-rated single depression item NPI-NH M (SD)	161	1.7 (2.8)
Informant-rated combined NPI-NH depression-anxiety score M (SD)	160	2.8 (4.5)
Quality of Life		
Self-rated QOL-AD, M (SD)	96	32.1 (5.5)
Informant-rated QUALIDEM, M (SD)	161	77.4 (14.3)
Dementia stage FAST, n (%)	161	
Mild dementia		5 (3)
Mid-stage dementia		2 (1)
Moderately severe dementia		135 (84)
Severe dementia		19 (12)
Functional Status		
Barthel Index score, M (SD)	161	53.6 (26.2)
Neuropsychiatric Symptoms		
Informant-rated NPI-NH, M (SD)	161	16.6 (16.3)
Psychotropic Medication		
Antidepressant present, n (%)	161	51 (32)

*M* Mean, *SD* Standard Deviation, *MMSE* Mini-Mental State Examination, *GDS-15* Geriatric Depression Scale, *NPI-NH* Neuropsychiatric Inventory—Nursing Home Version, *QOL-AD* Quality of Life in Alzheimer’s Disease, *QUALIDEM* Dementia specific quality of life instrument *FAST* Functional Assessment Staging

Self-rated reports of depressive symptoms (GDS-15) were obtained from 121 participants. Fourty-five PWD (37%) were classified as depressed (GDS-15 score ≥ 5), 69% of them were women. According to the GDS-15, 35 participants had mild, 8 had moderate, and 2 had severe depression [37]. Data from 161 participants with available informant-ratings of depressive symptoms were analyzed, using both the single depression item of the NPI-NH (*n* = 161) and the combined depression-anxiety (*n* = 160) items. A total of 63 participants (39%) were classified as depressed by the single depression item of the NPI-NH, 79% of them were women. There was a weak correlation between the single depression item of the NPI-NH and GDS-15 scores (*r* = 0.23, *p* = 0.01). A total of 21 participants (13%) were classified as depressed by the combined depression-anxiety items of the NPI-NH, 90% of them were women. We found no significant correlation between the combined depression-anxiety scores of the NPI-NH and GDS-15 scores (*r* = 0.13, *p* = 0.16).

Dementia stage assessed with FAST was higher in participants who were not able to give self-ratings of depressive symptoms compared to those who could (U = 1575.0, *p* < 0.001). For the 120 PWD with available self- and informant-rated data, a Kappa coefficient of 0.22 (*p* = 0.02) was calculated between GDS-15 and the single depression item of the NPI-NH, indicating a minimal level of agreement between both measures [61]. Table 2 shows the classification of all participants with available data on both screening instruments. No agreement was found between GDS-15 scores and the combined NPI-NH depression-anxiety classification (Kappa coefficient = 0.05; *p* = 0.47).

**Table 2:** Prevalence and classification of depression according to GDS-15 and single depression item of the NPI-NH, *n* = 120

		Classification according to single NPI-NH depression item		
		No depression	depression	Total
Classification according to GDS-15	No depression	53 (44%)	23 (19%)	76 (63%)
	Depression	21 (18%)	23 (19%)	44 (37%)
	Total	74 (62%)	46 (38%)	120

*GDS-15* Geriatric Depression Scale 15 items, *NPI-NH* Neuropsychiatric Inventory—Nursing Home Version

Daily intake of antidepressants was reported for 51 (32%) of the 162 PWD included in the study. While 41 of them were prescribed one antidepressant, 10 PWD took two antidepressants daily. No concordance of GDS-15 scores and antidepressant medication was found (Kappa coefficient = 0.03; *p* = 0.77), 36% of those classified as depressed and 33% of those not classified as depressed with GDS-15 received antidepressant treatment. As for classification by the single depression item of the NPI-NH, 38% of those with and 28% of those without depression received antidepressants, which also indicates no agreement (Kappa coefficient = 0.11; *p* = 0.16). Regarding the combined depression-anxiety classification, 38% of those with and 35% of those without depression received antidepressants, which also indicates no agreement (Kappa coefficient = 0.05; *p* = 0.51). Intake of a potentially inadequate drug was reported for 4 participants (2%).

Separate multivariate analyses were conducted for informant- and self-rated measures of depressive symptoms. For informant-rated depressive symptoms, multivariate analysis revealed higher informant-rated depressive symptoms rated with the single depression item from the NPI-NH were associated with lower informant-rated quality of life (β = -0.32; 95%CI: -0.52 to -0.13, *p* = 0.001) and lower dementia stage (β = -0.31; 95%CI: -0.52 to -0.10, *p* = 0.004), see Table 3. The combined informant-rated depression score using both the depression and anxiety items from the NPI-NH showed negative associations with self-rated quality of life (β = -0.12; 95%CI: -0.22 to -0.03, *p* = *0.0*1) and with dementia stage (β = -0.37; 95%CI: -0.67 to -0.07, *p* = 0.02) and a positive association with neuropsychiatric symptoms (β = 0.30; 95%CI: 0.10 to 0.51, *p* = 0.004). Higher self-rated depressive symptoms were associated with lower self-rated quality of life (β = -0.32; 95%CI: -0.45 to -0.19, *p* < 0.001), lower informant-rated quality of life (β = -0.25; 95%CI: -0.43 to -0.07, *p* = 0.005) and lower functional status (β = -0.16; 95%CI: -0.32 to -0.01, *p* = 0.04). Age, gender and antidepressant medication were not associated with self- or informant-rated depression, respectively. For the purpose of a sensitivity analysis, all of the reported tests of concordance and multivariate analyses were also conducted in the subgroup of participants without severe dementia (FAST level < 7; *n* = 142). These analyses yielded the same results, the only exception being that the association between self-reported depressive symptoms and functional ability failed to maintain statistical significance (β = -0.11; 95%CI: -0.30 to 0.08, *p* = 0.25).

**Table 3:** Multivariate associations of study variables with self- and informant-rated depression

	*Informant-rated depression*						*Self-rated depression*		
	*Single NPI-NH* *depression item* *n* = *161*			*Combined NPI-NH depression-anxiety score* *n* = *94*			*GDS-15* *n* = *95*		
Variable	β^a^	95% CI	*p*	β^b^	95% CI	*p*	β^b^	95% CI	*p*
Age	-.10	-.25 to .07	.29	-.11	-.22 to .01	.08	-.06	-.23 to .12	.51
Gender (0 = male, 1 = female)	-.11	-.44 to .23	.53	-.16	-.44 to .12	.27	.18	-.33 to .67	.49
Quality of Life (QOL-AD)	-.10	-.25 to .04	.15	**-.12**	**-22 to -.03**	**.01**	**-.32**	**-.45 to -.19**	** < .001**
Quality of Life (QUALIDEM)	**-.32**	**-.52 to -.13**	**.001**	-.28	-.61 to .06	.11	**-.25**	**-.43 to -.07**	**.005**
Dementia Stage (FAST)	**-.31**	**-.52 to -.10**	**.004**	**-.37**	**-.67 to -.07**	**.02**	-.12	-.32 to .08	.23
Functional status (Barthel Index)	.02	-.17 to .21	.84	-.01	-.15 to .13	.88	**-.16**	**-.32 to -.01**	**.04**
Neuropsychiatric symptoms (NPI-NH)	.09	-.17 to .35	.50	**.30**	**.10 to .51**	**.004**	.22	-.50 to .06	.13
Antidepressant (0 = not present, 1 = present)	-.09	-.40 to .21	.55	-.13	-.33 to .08	.23	.01	-.39 to .40	.97

*GDS-15* Geriatric Depression Scale, *NPI-NH* Neuropsychiatric Inventory—Nursing Home Version, *QOL-AD* Quality of Life in Alzheimer’s Disease, *QUALIDEM* Dementia specific quality of life instrument, *FAST* Functional Assessment Staging

Coefficients were estimated with linear mixed models^a^ and generalized estimating equations^b^. Clustering of measurements in nursing homes and participants was accounted for

## Discussion

In the present study, we investigated the agreement between two of the most frequently applied instruments for self- (GDS-15) and informant-rated (single depression item and combined depression-anxiety items of the NPI-NH) depressive symptoms in nursing home residents living with dementia. We also investigated factors associated with both screening measures and concordance of both modalities with antidepressant medication. In line with our hypothesis, we found minimal agreement between self- and single item informant-ratings. Contrary to our expectations however, the concordance between self- and informant-rated depressive symptoms did not improve with the combined depression-anxiety score. As expected, we found disparate association patterns for self- and informant-ratings with other study variables.

### Concordance of self- and informant-rated depressive symptoms

We found high prevalence of clinically relevant depressive symptoms measured with both self- and informant-rated measures. The prevalence of clinically relevant depressive symptoms found in our study was comparable for both modalities, with a slightly higher prevalence rate for single item informant-rated depressive symptoms (i.e., 37% for self-rated depressive symptoms and 39% for single item informant-rated depressive symptoms). This is in line with prevalence rates reported in previous studies [5, 21]. However, prevalance of the combined informant-rated depression-anxiety subsyndrome was 13%. Kwak et al. [28] also reported higher prevalence of non-clinically significant informant-reported depression compared to self-reported depression in PWD with mild dementia. However, as we expected, the concordance between both screening measures was only minimal. This may in part be attributed to disparities in the applied instruments. The GDS scale can only be answered by the person concerned. Therefore, the NPI is frequently used for external assessment. However, in contrast to the GDS, the NPI was not soley designed to assess depressive symptoms and may not measure the same symptoms assessed with GDS [6]. This may limit the comparability of these scales, and clinicians need to take this into consideration when using these instruments in nursing home residents with dementia.

Discrepancies in self- and informant measures of depression may also stem from deficits of PWD to rate their own mood due to impaired verbal expression and potential confounding with cognitive symptoms [62]. Informant-ratings are therefore frequently used to assess symptoms in PWD and are recommended to corroborate or substitute patients’ self-reports [63]. Research on prevalence of depression in nursing home residents with dementia usually relies on informant-reports, which are mostly obtained from clinicians or staff members such as trained nursing professionals [45]. However, deviations in self- and informant-ratings can reflect biases related to negative views of informants about PWD, increased caregiver burden or misinformation about depression in dementia, going along with a systematic overestimation of depression [64–66]. Furthermore, informant-ratings can only rely on observed behavior and become increasingly biased in PWD who can not adequately communicate how they are feeling due to pain, cognitive impairment or NPS such as apathy [5]. Therefore, future studies should focus on specific properties of self- and informant-ratings of depressive symptoms in PWD in order to identify and minimize inherent biases.

Not all participants in our study were able to give self-reports, and dementia stage was more advanced in those participants. In the course of dementia, progressing cognitive impairment is a known challenge. Self-reports can lead to excessive demands in people with severe dementia which makes it difficult for them to give accurate reports of their conditions. Balsamo et al. [67] recently reviewed self-report measures for assessment of depressive symptoms in older adults and concluded that many of the available screening instruments (e.g., Becks Depression Scale-II, Center for Epidemiological Studies Depression Scale, Zung Self-rating Depression Scale) do not sufficiently consider cognitive or sensory impairments among older patients. Although the GDS was specifically developed for geriatric patients and contains less somatic items than other screening measures, the validity of the GDS can be diminished in people with moderate to severe dementia [21, 68]. Therefore, informant-rated screening tools such as the depression item of the NPI-NH are frequently used and may offer a pragmatic approach for widespread depression diagnosis in everyday nursing home settings [69]. However, as the NPI-NH was not specifically designed to measure depression, it may not be sufficient to capture depressive symptoms alone. Therefore, whenever possible, it is important to ascertain the views of PWD, as there can be large discrepancies between staff and PWD depression ratings [70]. Furthermore, cognitively impaired patients can underreport symptoms, while caregivers have been known to overreport [6]. PWD differ in their level of insight and deterioration of a sense of self [71]. Self-reports strengthen and support autonomy in PWD and allow them to express their own views [32, 72]. Snow et al. [73] found that an existing diagnosis of dementia per se does not lead to inaccurate self-reports of depression. Perfect et al. [74] investiged challenges and benefts of using self-reports in research with PWD. They conclude that self-reports can and should be applied in studies with people in all stages of dementia. Our findings underscore the importance of incorporating self-reports when assessing depressive symptoms in PWD, as some internalized depressive symptoms such as feelings of worthlessness, guilt and loneliness may not be evident to external informants [36, 45].

### Factors associated with both self- versus informant-rated depressive symptoms

Both measures showed divergent association patterns with other study variables. In line with our expectations, higher self-rated depressive symptoms were associated with lower self-rated quality of life and lower fuctional abilty, whereas higher informant-rated single item depressive symptoms were associated with lower informant-rated quality of life. Our results confirm the widely reported link between depression and quality of life in PWD [35]. We observed a method factor for both modalities showing associations of self- and informant-measures of depressive symptoms and quality of life, respectively. There was also an unexpected crossover from self-reported depressive symptoms to informant-reported quality of life: Higher self-reported depressive symptoms was related to lower informant-rated quality of life. Moreover, higher informant-rated depression-anxiety scores were associated with lower self-rated quality of life, indicating that PWD with both clinically relevant depressive and anxiety symptoms may feel especially distressed. These results may be partly contributed to the scales used for assessment of quality of life. Informant-ratings were collected with the more extensive QUALIDEM instrument, in order to minimize self-report burden in participating PWD. However, the conceptual differences may also affect comparability of the scales.

In line with previous findings [75, 76], we found depressive symptoms to be lower in participants with higher dementia severity. However, this was only true for informant-rated depression. In contrast, self-rated depression was related to lower functional status. Knapskog et al. [77] found impaired activities of daily living (ADL) were clearly associated with depression in PWD, and Crespo et al. [78] found functional autonomy to be a main predictor of informant-rated quality of life in nursing home residents with dementia. Taken together, our results suggest that depression may go underrecognized by staff informants in residents with higher stages of dementia and greater levels of functional impairment, as their focus may shift to other symptoms [12]. In this regard, we found that PWD with higher combined informant-rated depression-anxiety scores also had more informant-rated neuropsychiatric symptoms. Furthermore, staff informants may not always recognize depression in residents with severe dementia due to the overlap of depression and dementia symptoms [68, 79]. This could lead to an undersupply of therapeutic interventions in PWD with moderate to severe dementia. It has been recommended that intervention strategies should be applied in all stages of dementia, as the prevalence of depressive symptoms does not seem to be linked to the severity of dementia [69] and there is solid evidence showing that depression in dementia is treatable [20, 21]. Non-pharmacological treatments such as cognitive-behavioral therapy, interpersonal therapy or multimodal interventions are effective in PWD and should be used as first choice interventions in nursing home residents with dementia [21, 80]. This is also in line with a person-centered approach in dementia care, which builds around the indivdual needs of PWD, and has found to be essential for high quality dementia care [81, 82]. For example, based on data from randomized controlled studies, a systematic review and meta-analysis reported positive effects of person-centered care on agitation, neuropsychiatric symptoms, depression and quality of life for PWD [83]. Another study evaluating person-centered care for PWD in acute inpatient care showed, among others, positive effects of the intervention on well-being, agitation, and quality of life [84]. Nursing home staff should receive extensive training on detection of depression in dementia, in order to distinguish between overlapping symptoms of depression and dementia [85]. Taken together, the unique association patterns of self- and informant-rated depression with other variables indicate that depression has multidimensional aspects, underlining the importance of multimodal approaches in order to reach an accurate and comprehensive assessment of depressive symptoms in PWD [65].

### Concordance of self- and informant-rated depressive symptoms with antidepressant medication

In line with Kwak et al. [28], we found no significant agreement of either screening instrument with antidepressant medication. Descriptive review of antidepressant pharmacotherapy revealed the occasional use of potentially inadequate drugs in some participants. Although there is a growing body of research indicating that pharmacological therapy of depression in PWD is essentially ineffective and potentially harmful [86], it remains unclear if these empirical findings are known and considered in real world medical settings. In line with findings reported by Kramer et al. [27], our data show that a large proportion of participants received antidepressants, even though there were no apparent self- or informant-rated depressive symptoms. Although antidepressants such as selective serotonin reuptake inhibitors (SSRIs) are frequently prescribed for managing depressive symptoms in dementia [87], two recent systematic reviews of pharmacological treatment of depression have reported inconclusive evidence on the efficacy of antidepressants in PWD [86, 88]. Furthermore, there is a consensus among evidence-based treatment guidelines for dementia that pharmacological options should only be considered if non-pharmacological options have failed, in order to avoid unwanted side-effects [20]. Due to the cross-sectional nature of our data, we cannot rule out that the medication was initially prescribed due to a pre-existing depression disorder. However, in light of the mixed findings on pharmacological treatment of depression, medication should be evaluated in a timely fashion and if possible reduced upon remission.

## Limitations

Although our study has many strengths, some important limitations must be mentioned. Our main objective was to investigate the concordance and association patterns of self- and informant-rated depressive symptoms in nursing home residents in a cross-sectional design. Future studies should include longitudinal and factorial designs in order to shed more light on specific properties of self- and informant-ratings. A further limitation is the absence of a structured clinical interview as gold standard method for diagnosis of depression. Both instruments used in our study (GDS-15 and NPI-NH) were designed for depression screening and can in no way substitute a full diagnostic workup conducted by trained medical experts such as clinical psychologists or physicians. Future studies should take a more comprehensive diagnostic approach to address discrepancies between self- and informant assessments and clarify whether they are systematic in nature and due to the fact that different facets of depression are being assessed, or whether they are in fact unsystematic and due to the limited reliability and validity of the measurement instruments. Innovative measurements such as Ecological Momentary Assessments may also provide further insights into fluctuating affective states, which have been reported in PWD. Finally, the GDS-15 is not recommended for patients with severe dementia and the overall usefulness is uncertain in PWD with MMSE-scores lower than 10 [52]. This limits the validity of our findings, as some participants may not have been able to give accurate self-reports due to cognitive or functional limitations. Other instruments such as the CSDD which combines self- and clinician-ratings [40] may be more suited for patients with advanced dementia. Moreover, we used two different instruments to measure agreement of informant- and self-rated depression. Although we chose an inclusive approach and aimed to assess self-ratings from all participants, we cannot rule out that the heterogeneity of the applied self- and informant-rated assessment tools for depressive symptoms and quality of life, which are partly based on different concepts, may limit the validity and generalizability of our results. Future studies should utilize informant- and self-rating versions of the same instrument, in order to rule out instrument bias as compared to type-of-rating bias, which would provide valuable insights for clinical practice in nursing homes.

## Conclusion

Overall, the present study found low concordance of self- and informant rated depression and disparate association patterns with other variables. Clinicians should be aware of the potential imbalance between self- and informant-ratings of depressive symptoms in PWD. While informant-ratings are feasible is all dementia stages, they can only be used to assess visible symptoms displayed on the behavioral level. Insights about inner states, such as feelings of worthlessness and guilt need to be derived from self-reports. Therefore, self-reports are an essential part of depression diagnosis and an effort should be made to obtain self-reports from PWD regardless of their dementia stage. Adequate treatment should be offered to those affected by depression in a timely fashion, regardless of dementia severity and regular evaluation of prescribed antidepressants should be conducted, in order to avoid potential inappropriate use of antidepressants in nursing home residents with dementia.

## Data Availability

The datasets used and analysed are stored in a non-publicly available repository and are available from the corresponding author on reasonable request.

## Abbreviations

95%CI: 95% Confidence interval
ADL: Activities of Daily Living
AES-I: Apathy Evaluation Scale – Informant Version
cRCT: Cluster-randomised controlled trial
CSDD: Cornell Scale for Depression in Dementia
FAST: Functional Assessment Staging
GDS: Geriatric Depression Scale
GEE: Generalized Estimating Equations
LMM: Linear mixed-effects models
M: Mean
MADRS: Montgomery Asberg Depression Rating Scale
MMSE: Mini Mental State Examination
NPI: Neuropsychiatric Inventory
NPI-NH: Neuropsychiatric Inventory—Nursing Home Version
NPS: Neuropsychiatric Symptoms
PWD: People with dementia
QOL-AD: Quality of Life in Alzheimer’s Disease
REML: Restricted maximum likelihood estimation
SD: Standard deviation
SSRIs: Selective serotonin reuptake inhibitors
